# Unravelling the challenges of mycetoma: a case series highlighting diagnostic dilemmas and therapeutic triumphs

**DOI:** 10.1093/skinhd/vzae017

**Published:** 2025-02-14

**Authors:** Mahesh Mathur, Neha Thakur, Sunil Jaiswal, Sandhya Regmi, Supriya Paudel

**Affiliations:** Department of Dermatology, Venereology, and Leprology, College of Medical Sciences and Teaching Hospital, Bharatpur, Nepal; Department of Dermatology, Venereology, and Leprology, College of Medical Sciences and Teaching Hospital, Bharatpur, Nepal; Department of Dermatology, Venereology, and Leprology, College of Medical Sciences and Teaching Hospital, Bharatpur, Nepal; Department of Dermatology, Venereology, and Leprology, College of Medical Sciences and Teaching Hospital, Bharatpur, Nepal; Department of Dermatology, Venereology, and Leprology, College of Medical Sciences and Teaching Hospital, Bharatpur, Nepal

## Abstract

Mycetoma is a chronic suppurative granulomatous infection of the deep dermis and subcutaneous tissue prevalent in tropical and subtropical regions and is caused by filamentous aerobic bacteria (actinomycetoma) or true fungi (eumycetoma), representing 60% and 40% of cases worldwide, respectively. The causative organism enters into the subcutaneous tissue, usually of the foot, from contaminated soil or vegetative material through inoculation from a thorn prick, or repeated trauma. It commonly affects men, farmers and field workers. Differentiating eumycetoma from actinomycetoma can be challenging but is required before starting prolonged treatment. One of our patients presented with lesions on the thigh and in a sporotrichoid pattern that is atypical, while the other two patients were treated with antifungal medication for eumycetoma for years without proper investigation and improvement. Early diagnosis of actinomycetoma is mandatory to prevent tissue destruction, bone invasion and ultimate loss of function by proper investigative workup, histopathology and direct microscopy of discharge. We here report three cases of actinomycetoma with clinical and microbiology profiles treated successfully with tablets of trimethoprim-sulfamethoxazoleand amoxicillin–clavulanic acid along with folic acid as proposed by the Cochrane systemic review protocol 2018.

## Teaser

Mycetoma is a chronic suppurative granulomatous infection of the deep dermis and subcutaneous tissue prevalent in tropical and subtropical regions and is caused by filamentous aerobic bacteria (actinomycetoma) or true fungi (eumycetoma), representing 60% and 40% of cases worldwide respectively. Differentiating eumycetoma from actinomycetoma can be challenging but is required before starting prolonged treatment. Early diagnosis of actinomycetoma is mandatory to prevent tissue destruction, bone invasion and ultimate loss of function by proper investigative work-up, histopathology and direct microscopy of discharge. We here report three cases of actinomycetoma with clinical and microbiology profiles treated with tablets of trimethoprim-sulfamethoxazole, amoxicillin–clavulanic acid.

**What is already known about this topic?**

- Mycetoma is a neglected disease prevalent in tropical and subtropical regions with a chronic course.
- Two forms of mycetoma have been identified (i.e. eumycetoma and actinomycetoma) and are caused by fungi and bacteria, ­respectively.
- Patients present with a triad of painless subcutaneous swelling, multiple sinus tracts and discharge-containing grains.
- These grains are pathognomic.
- As it has a chronic course, longer-term therapy is mandatory.
- Young men and farmers are commonly affected.

**What does this study add?**

- Differentiating actinomycetoma from eumycetoma is challenging.
- When actinomycetoma is suspected, additional simple techniques like Gram stain, AFB stain, 10% KOH mount and PAS stain can be performed, the results of which can be rewarding as observed in our patients who were previously being treated for eumycetoma.
- All of our patients responded to Cochrane Collaboration review protocol 2018 treatment guidelines, which is worth discussing.
- We have included the follow-up photographs of our patients.

##  

Mycetoma is a persistent granulomatous infection affecting the deep dermis, subcutaneous tissue, fascia and even bone. It is commonly found in tropical and subtropical regions and can be attributed to filamentous aerobic bacteria (actinomycetoma) or fungi (eumycetoma).^1^ It was first recognized by Dr John Gill in 1842 in Madurai, India, and was hence termed ‘Madura foot’ later on in 1844.^2^ Mycetoma was added to the list of neglected tropical diseases by the World Health Organization in 2016.^3^ The causative organism enters into the subcutaneous tissue, usually of the foot, from contaminated soil or vegetative material through a penetrating injury, thorn prick or repeated trauma. It commonly affects men, farmers and field workers of low socioeconomic status who work barefooted in fields. The classical clinical triad consists of painless subcutaneous swelling, multiple sinus tracts and discharge-containing grains that vary in size, colour and consistency, representing the microorganismal colonies.^4^ Various diagnostic techniques for identifying the organism include culture from grains, surgical biopsy and histopathological examination, fine needle aspiration cytological examination and polymerase chain reaction.^5^ We report three cases with microbiology profiles of actinomycetoma in patients attending the College of Medical Sciences, Nepal.

## Case reports

### Patient 1

**Figure 1:**
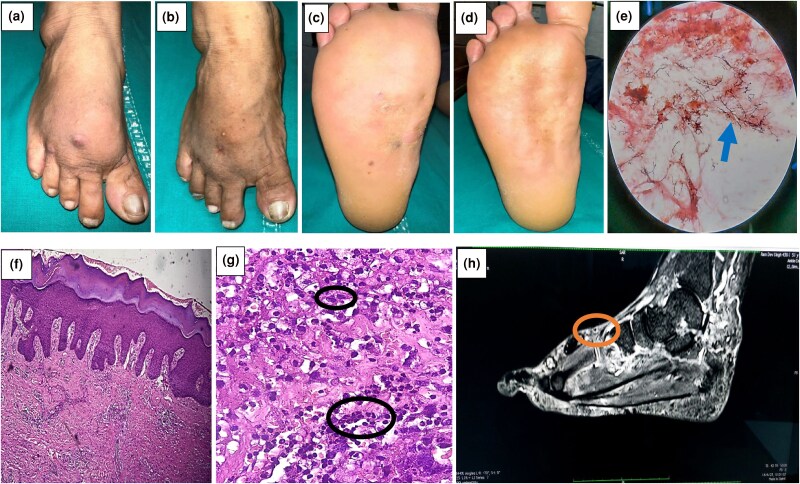
(a) Erythematous nodules and diffuse swelling on the dorsum of right foot. (b) Nodules healed and swelling subsided after 1 year of treatment. (c) Scaly nodules and plaques on the plantar aspect of the right foot. (d) Follow-up image with improvement after 1 year of treatment. (e) Gram stain smear shows many Gram-positive filamentous branching bacilli (blue arrow). (f) Histopathological image (haematoxylin and eosin, ×10) shows change in the epidermis. (g) Neutrophilic collection and histiocytes in the mid and lower dermis at ×40 (circles). (h) Dot-in-circle sign (circle) on magnetic resonance imaging.

A 50-year-old man from the Terai region of Nepal presented with painless swelling, multiple nodules and recurrent draining sinuses on the plantar and dorsal aspects of the right foot for the past 6 years. Nodules ranged in size from 0.5 to 1 cm in diameter (Figure 1a, c). The condition was misdiagnosed as eumycetoma and treated with antifungal medication (itraconazole 200 mg twice daily) for 5 years at another centre without proper investigations or improvement. His liver enzymes were twice the upper limit of normal. Gram staining at our centre showed many Gram-positive filamentous branching bacilli (Figure 1e). Excisional deep biopsy from one of the nodules showed white-coloured grains and histopathological examination revealed pseudoepitheliomatous hyperplasia and dermal inflammatory cell infiltrates composed of neutrophils and histiocytes (Figure 1f, g). Periodic acid–Schiff (PAS) and acid-fast bacilli (AFB) stains were negative. Magnetic resonance imaging (MRI) revealed a ‘dot-in-circle’ sign and routine blood investigations were normal (Figure 1h). As histopathology failed to provide the diagnosis, actinomycetoma was diagnosed based on microbiology and imaging. He had sensorineural hearing loss confirmed by audiometric testing, and therefore, as per the Cochrane systematic review on actinomycetoma in 2018, he received oral trimethoprim–sulfamethoxazole (960 mg) and amoxicillin–clavulanic acid (1 g) twice daily, and folic acid 5 mg once daily (Table 1).^3^ He showed significant clinical improvement at the 1-year follow-up and has not been under treatment for last 6 months, without recurrence of skin lesions (Figure 1b, d).

**Table 1:** Summary of the clinical and demographic features of three patients with mycetoma

Patient	Age	Sex	Site	Duration of symptom	Treatment	Duration of treatment	Outcome
1	50 years	Male	Right foot	6 years	Oral trimethoprim–sulfamethoxazole (960 mg), amoxicillin–clavulanic acid (1 g) twice daily, and folic acid 5 mg once daily	1 year	Clinical resolution No recurrence over 6 months of follow-up
2	27 years	Male	Right thigh	1 year	Oral trimethoprim-sulfamethoxazole (960 mg), amoxicillin–clavulanic acid (1 g) twice daily, and folic acid 5 mg once daily	6 weeks	Healed sinuses and decreased swelling
3	78 years	Female	Right foot	15 years	Oral trimethoprim–sulfamethoxazole (960 mg), amoxicillin–clavulanic acid (1 g) twice daily, and folic acid 5 mg once daily	4 months	Decreased foot swelling and pain Healed sinuses and crusted nodules

### Patient 2

**Figure 2:**
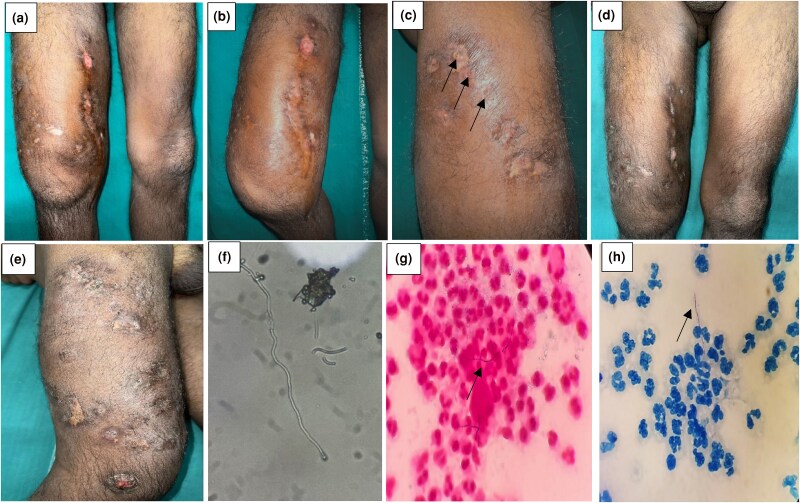
(a, b) Diffuse swelling of right thigh, ulceration and scarring. (c) Multiple discharging sinuses (black arrows) and healed sinuses on the lateral and posterior aspect of right thigh. (d, e) Images at 6 weeks of follow-up. (f) KOH examination shows filamentous bacilli. (g) Gram stain shows Gram-positive filamentous branching bacilli along with neutrophils (arrow). (h) Acid-fast bacilli stain shows acid-fast filamentous bacilli along with neutrophils (arrow).

A 27-year-old man presented with diffuse painless swelling and multiple sinuses discharging purulent material and pale grains on the right thigh for over a year (Figure 2a–c). A few lesions had healed with scarring and he developed a solitary nonhealing ulcer on the medial aspect of his right thigh over the last 3 months (Figure 2d, e). The 10% potassium hydroxide (KOH) mount showed filamentous bacilli and Gram staining revealed positive filamentous branching bacilli along with neutrophils, which were acid-fast on AFB staining (Figure 2f–h). The histological analysis revealed epidermal hyperplasia, dermal inflammatory cells with predominant neutrophils, lymphocytes, histiocytes and eosinophils, along with neutrophilic abscess and multinucleated giant cells. Routine investigations, X-ray and MRI of the right thigh were normal. Significant clinical response was seen at the 6-week follow-up after treatment with medications as recommended by the Cochrane protocol for actinomycetoma, and he remains under treatment (Table 1).

### Patient 3

**Figure 3:**
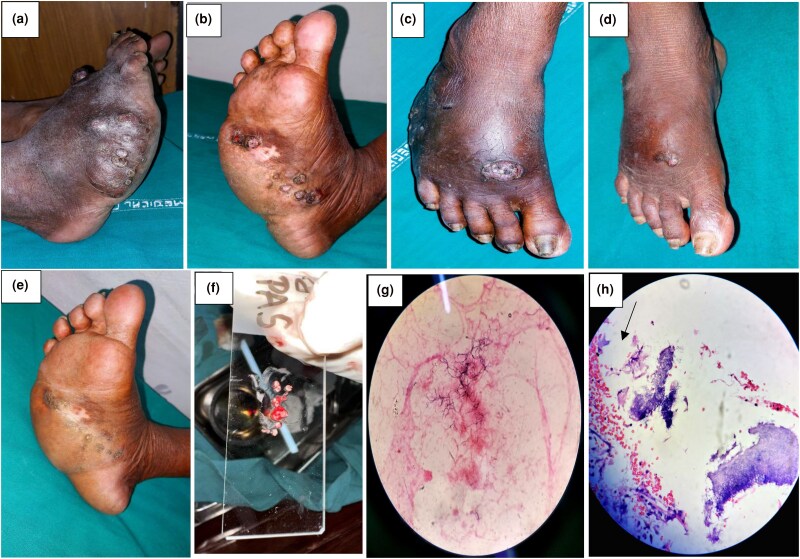
(a, b) Multiple erythematous and crusted nodules on the dorsolateral surface, medial plantar aspect and sole of the right foot surrounded by fluctuant nontender swelling, solitary, crusted and hyperkeratotic mass discharging white grains on the dorsum of right foot. (c–e) Follow-up images at (c) 2 months and (d, e) 4 months. (f) Pale-coloured grains taken from discharge. (g) Multiple branching filamentous Gram-positive rods on Gram stain. (h) Histopathology at ×40 revealing clumps of filamentous bacteria.

A 78-year-old woman with hypertension and diabetes presented with multiple erythematous and crusted nodules on the right foot and a solitary, crusted, hyperkeratotic mass discharging white grains on the dorsum surrounded by fluctuant ­nontender swelling and hyperpigmentation for approximately 15 years (Figure 3a–c). She had history of recurrent minor trauma while working in the field and multiple incisions and drainage were done for the swelling at nearby primary health centres. She had previously taken itraconazole capsules on and off for eumycetoma. We performed Gram staining, which showed multiple branching filamentous Gram-positive rods, and histopathology revealed fibrocollageneous dermis with inflammatory infiltrates and clumps of filamentous bacteria (Figure 2f–h). PAS and AFB stains were negative. She received a Cochrane regimen of antibiotics and her 2- and 6-month follow-up results were satisfactory (Figure 3c–e) (Table 1).

## Discussion

Actinomycetoma commences with traumatic inoculation of the causative agent from contaminated soil and plants; therefore, infection is often localized to the foot (80%) in young men, farmers and labourers. However, the upper limb (9%), trunk (4%), head and neck (4%) and thigh and knee (3%) may be involved.^6^ Initially, a small, painless nodule appears at the site of injury, which over time softens and ulcerates, discharging characteristic granules. Grains result in progressive infiltration of tissues, involving fascia, muscles and bone.

When actinomycetoma is suspected, additional simple techniques like Gram and AFB staining, 10% KOH mounting and PAS staining can be performed, the results of which can be rewarding, as observed in our patients who were being treated for eumycetoma over a long period. MRI provides a comprehensive assessment of bone and soft tissue involvement with a diagnostic dot-in-circle sign.^4^ Histopathology, despite its failure in diagnosing the first two cases, was successful in diagnosing the third case as clumps of bacteria were easily demonstrated. Differential diagnoses of mycetoma include cutaneous tuberculosis, especially gumma of the foot, other atypical mycobacteria, blastomycosis, sporotrichosis, botryomycosis, soft tissue tumours and chronic osteomyelitis. However, grains are pathognomic for mycetoma.^1,7–10^

Our second patient presented with lesions on the thigh and in a sporotrichoid pattern, which is a rare presentation of mycetoma and a good reminder that this disease can also affect areas other than the foot. Similarly, the first patient developed drug-induced hepatic injury due to an incorrect diagnosis and prolonged treatment with itraconazole. Proper investigative workup and histopathology guided by clinical correlation, and direct microscopy of discharging grains need to be performed for early diagnosis and the prevention of complications. Immediate treatment for a prolonged duration is mandatory as relapse follows shorter courses of medication. We started our patients with tablets of trimethoprim–sulfamethoxazole 960 mg twice daily, amoxycillin and clavulanic acid 1 g twice daily, and folic acid 5 mg as per the Cochrane systematic review on mycetoma in 2018.^3^ The ‘Welsh regimen’ has co-trimoxazole with parenteral amikacin in one to four 5-week cycles.^11^ The ‘Ramam two-step regimen’ replaced amikacin with the alternative of gentamicin in the intensive phase, i.e. co-trimoxazole and gentamicin for 4 weeks followed by a maintenance phase of co-trimoxazole and doxycycline for 5–6 months.^12^ The need for regular monitoring of renal function and audiometric testing with aminoglycosides, the requirement of repeated hospital admission for parenteral application, the high cost of medications, poor compliance, associated comorbidities, and lack of education of patients in our part of the world made us select the Cochrane Collaboration review protocol 2018 and the results thus observed are significant.

In summary, these cases emphasize the challenges of diagnosing and treating mycetoma, highlighting the importance of proper diagnostic techniques, histopathological examination and adherence to treatment protocols for favourable outcomes.

## Data Availability

The data underlying this article will be shared on reasonable request to the corresponding author.
